# Hyperbaric oxygen therapy for painful bladder syndrome/interstitial cystitis resistant to conventional treatments: long-term results of a case series in Japan

**DOI:** 10.1186/1471-2490-11-11

**Published:** 2011-05-24

**Authors:** Tomoaki Tanaka, Yujiro Nitta, Kazuya Morimoto, Noriaki Nishikawa, Chikako Nishihara, Satoshi Tamada, Hidenori Kawashima, Tatsuya Nakatani

**Affiliations:** 1 Department of Urology, Osaka City University Graduate School of Medicine, Osaka, Japan

## Abstract

**Background:**

There is no confirmed strategy for treating painful bladder syndrome/interstitial cystitis (PBS/IC) with unclear etiology. Therefore, a pilot study was carried out to evaluate the efficacy and safety of hyperbaric oxygen (HBO) therapy in treatment-resistant PBS/IC patients.

**Methods:**

HBO treatment (2.0 ATA for 60 minutes/day × 5 days/week for 2 or 4 weeks) was performed on 11 patients with severe symptoms that had not been improved by previous therapy regimens between December 2004 and July 2009.

**Results:**

Seven of the 11 patients demonstrated persistent improvement in symptoms during the 12 months after HBO treatment. These responders demonstrated a decrease in the pelvic pain scale and urgency scale from 7.7 ± 1.0 and, 6.6 ± 0.9 to 3.4 ± 2.5 and 4.3 ± 2.4 after 12 months, respectively (p < 0.05). The total score of the interstitial cystitis symptom index and 24-hour urinary frequency demonstrated a significant sustained decrease from the baseline. Two responders, who received an additional course of HBO 12 and 13 months after initial treatment, respectively, did not suffer impairment for more than two years. There was one case of transient eustachian tube dysfunction and three cases of reversible exudative otitis media as a consequence of HBO treatment.

**Conclusions:**

HBO is a potent treatment for PBS/IC patients resistant to conventional therapy. It was well tolerated and provided maintained amelioration of pain, urgency and urinary frequency for at least 12 months.

## Background

Painful bladder syndrome/interstitial cystitis (PBS/IC) is a collective term covering a range of clinical complaints and pathological findings. Approximately 10-50% of PBS/IC patients demonstrate a classical mucosal ulcer (Hunner's ulcer), and the majority are diagnosed on the basis of positive factors and exclusions derived from the diagnostic criteria of the National Institute of Diabetes and Digestive and Kidney Diseases for IC [1,2]. The etiology of PBS/IC includes a diversity of factors and remains poorly understood. Therefore, appropriate therapy has not been established from clinical evidence [3-5]. Hyperbaric oxygen (HBO) therapy has been reported to be effective in patients with cyclophosphamide-induced hemorrhagic cystitis and chronic radiation cystitis for approximately 20 years [6-9]. The pathological finding of chronic radiation cystitis is similar to PBS/IC, focusing on ischemia and a reduction in bladder capacity due to fibrosis of the bladder wall [10-12]. On the basis of these findings, a pilot study concerning HBO treatment in several PBS/IC patients whose symptoms had not been improved by other conventional treatments was carried out.

## Methods

From December 2004 to July 2009, 11 PBS/IC patients whose symptoms were resistant to conventional therapy were treated with HBO therapy. All patients had undergone conventional treatments including oral medication, intrasvesical instillation of heparin and hydrodistension. The ethical review board of our institute approved the study, and informed consent was obtained from all patients. Patients were treated with HBO (2.0 ATA for 60 minutes/day × 5 days/week for two or four weeks) sequentially after previous hydrodistention. After 10 sessions had been performed, patients were assessed and 10 more sessions were performed in some cases; eight patients underwent 10 sessions and three received 20 sessions (Table 1). In the case of patients with severe urgency or incontinence, pads were worn during treatment sessions. The efficacy of HBO treatment for PBS/IC disease was assessed using the score of O'Leary-Sant IC symptom and problem index (ICSI), comprising eight questions with the ranges of 0-5 and 0-4 with regards to pain and voiding symptoms, respectively, the scales of pelvic pain and urgency using a visual analogue scale (VAS) with the range 0-9, bladder capacity, daily voiding frequency, and endoscopic findings. A responder was defined as a patient with an improvement in ≥ 1 fraction among the total score of ICSI, and the scale of pain or urgency on VAS. The population of two related samples could not be assumed to be normally distributed. Therefore, statistical comparisons were performed using the Wilcoxon signed-rank test for changes from the baseline in the aforementioned parameters. P < 0.05 was considered to denote a statistically significant difference.

**Table 1:** Characteristics of HBO-treated patients and outcome of HBO

Patient	Sex	Age(years)	Symptom duration (years)	Bladder capacity on FVC (ml)	Times of hydrodistension before HBO	Sessions of HBO treatment	Response to HBO	PBS/IC subtype based on hydrodistension	Observation duration after HBO (months)
1*	female	79	2.2	40	2	20	Responder	Ulcerative	50

2*	female	61	4.5	60	2	20	Responder	Ulcerative	44

3	female	28	1.8	80	1	10	Non-responder	Non-ulcerative	4

4	female	68	2.4	70	4	10	Responder	Ulcerative	33

5	female	57	3.6	80	4	10	Responder	Ulcerative	15

6	male	65	4	50	2	20	Non-responder	Non-ulcerative	30

7	female	70	3.2	100	1	10	Responder	Ulcerative	14

8	female	50	2.1	90	2	10	Non-responder	Non-ulcerative	4

9	female	70	6.4	40	6	10	Responder	Ulcerative	13

10	female	43	3.5	120	1	10	Non-responder	Ulcerative	3

11	female	70	3.2	50	4	10	Responder	Ulcerative	12

*Two patients of case 1, 2 underwent additional 10 sessions after 13, 14 months, respectively.

## Results

**Figure 1:**
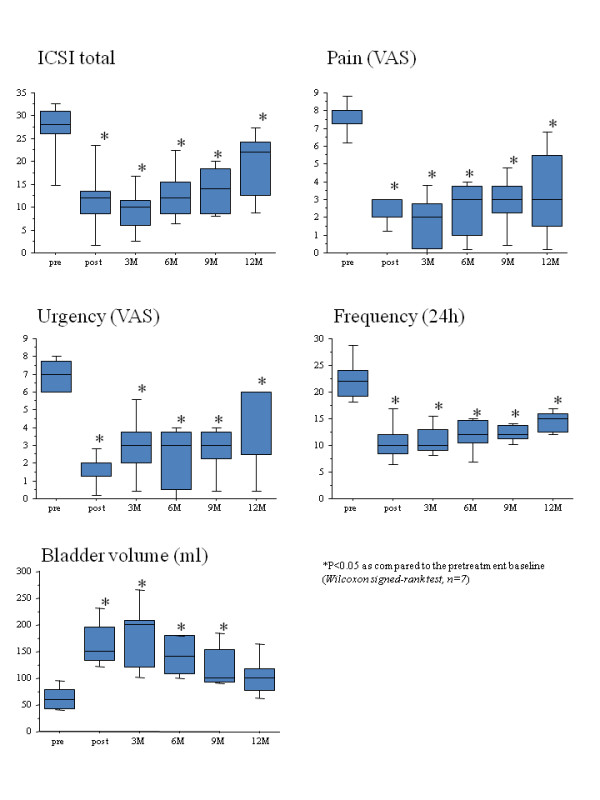
**Changes in evaluated parameters (the total score of ICSI, the scale of pain and urgency on VAS, 24-hour voiding frequency and maximum bladder volume) from the baseline in seven responders after three, six, nine, and 12 months follow-up after HBO treatment**. The bottom and top of the box are the lower and upper quartiles, respectively, and the ends of whiskers represent the minima and maxima of the samples.

**Figure 2:**
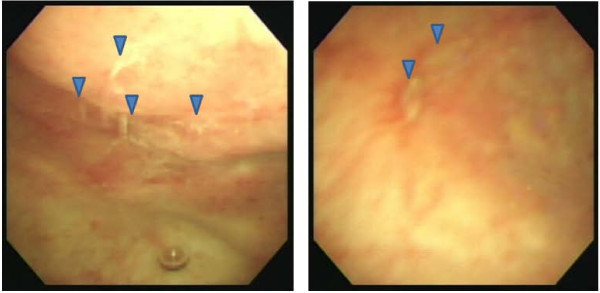
**Granulation of an ulcer in the bladder mucosa of an HBO-responder; the arrows indicate the lesions**.

The patients comprised 10 females and one male; the mean age was 60.0 years (range 28-79 years). The PBS/IC diseases in these 11 patients included eight cases of ulcerative type and three of non-ulcerative type, according to intravesical endoscopic findings (Table 1). Patients were followed up for a median period of 14 months (range 3-50 months) after HBO therapy. Seven of the 11 patients were classed as responders. Four patients, who demonstrated no remission or short-term improvement, were considered non-responders. Three of four non-responders had non-ulcerative endoscopic findings (Table 1). At the end of the HBO sessions, seven responders demonstrated a significant improvement in symptoms compared to the pre-treatment baseline (p < 0.05), and had sustained amelioration with mild impairment during the following 12 months (Figure 1). After 12 months, the scales concerning pelvic pain and urgency were still decreased from 7.7 ± 1.0 and 6.6 ± 0.9 to 3.4 ± 2.5 and 4.3 ± 2.4, respectively (p < 0.05). The total score of ICSI decreased from 26.7 ± 7.0 to 18.7 ± 7.4 (p < 0.05), and the 24-hour voiding frequency decreased from 22.4 ± 4.0 to 14.6 ± 2.0 (p < 0.05). Two patients (cases one and two) in the responder group, who had received 20 sessions at the time of the initial report, underwent 10 secondary sessions of HBO treatment 13 and 14 months after initial HBO therapy, respectively. The symptoms in these patients remained stable for more than two years. In addition, cystoscopic examination demonstrated marked granulation of the ulcerative lesion (Figure 2) at the end of HBO treatment in all responders. With regards to adverse events, there was transient eustachian tube dysfunction in one case and reversible exudative otitis media in three cases. However, no patients discontinued HBO treatment because of these side effects.

## Discussion

The mechanism of action underlying HBO treatment is attributed to hyper-saturation of the plasma with dissolved oxygen. This gives rise to an increased concentration gradient between the circulation and surrounding tissues, allowing oxygen to enter damaged hypoxic urothelial tissues. HBO treatment accelerates growth of healthy granulation in injured tissues via stimulation of leukocytic functions including phagocytosis and production of growth factors related to angiogenesis [13,14]. HBO therapy has been used predominantly for chronic radiation cystitis and cyclophosphamide-induced hemorrhage cystitis in the last 20 years [6-9]. Chronic radiation cystitis is characterized by various histological alterations including sub-mucosal hemorrhage, interstitial fibrosis and smooth muscle fibrosis [10], which correspond to classical PBS/IC with ulcerative lesions [11,15]. Therefore, it was hypothesized that HBO could be an effective treatment for PBS/IC with typical histological changes (glomerulations, Hunner's ulcer and interstitial fibrosis). Seven of 11 cases treated with HBO demonstrated a significant decrease in urinary frequency and pelvic pain and an increase in bladder capacity. Cystoscopic examination revealed the scarring or healing phase of ulcerative lesions in all responders. Furthermore, the positive effects on symptoms were sustained for a minimum of 12 months. Van Ophoven *et al*. carried out a pilot study concerning HBO in six PBS/IC patients [16]. Our group reported that HBO treatment resulted in a marked improvement of severe PBS/IC symptoms in the initial two cases [17]. In addition, van Ophoven's research group reported the effectiveness of HBO for PBS/IC on the basis of a randomized, double-blind, sham controlled clinical study [18]. This study revealed that the scale of pelvic pain in the HBO treatment group was significantly better than in the sham control group, and the amelioration in responders was sustained 12 months after HBO treatment. The results of our study are almost compatible with their report. Interestingly, secondary HBO treatment prolonged the period of remission in two cases (cases 1, 2). Therefore, it is likely that a repeated course of HBO could accelerate the healing phase of ulcerative PBS/IC disease. Three of the four cases that responded poorly to HBO presented with non-ulcerative PBS/IC. Thus, we speculate that ulcerative lesion with the most evident expression of bladder ischemia may be a predictive factor to result in good response to HBO. HBO therapy was well tolerated by patients; adverse events including visual disturbance, eustachian tube dysfunction and claustrophobia were unusual [19]. Furthermore, the advantage of HBO treatment over conventional therapies such as hydrodistension [15], intravesical instillation of dimethyl-sulfoxide (DMSO) [20] and intravesical submucosal injection of Botulinum toxin type A [21,22] is that it is non-invasive.

## Conclusions

The long-term efficacy of HBO treatment in 11 PBS/IC patients resistant to other conservative therapies was investigated. Seven of 11 patients, who underwent 10 or 20 sessions of HBO treatment, demonstrated good amelioration of the evaluated parameters including IC symptom score, scale for pain and urgency, 24-hour urinary frequency and bladder volume, for at least one year. Furthermore, two responders with worsening symptoms experienced prolonged improvement after additional HBO treatment sessions. HBO therapy was well tolerated, with few patients developing transient eustachian tube dysfunction and reversible exudative otitis media.

The present study suggests that HBO could be used for the treatment of PBS/IC patients resistant to various conventional therapies.

## List of abbreviations

FVC, frequency volume chart; pre, pre-HBO treatment; post, post-HBO treatment; HBO, hyperbaric oxygen; PBS/IC, painful bladder syndrome/interstitial cystitis; ATA, atmosphere absolute; ICSI, Interstitial Cystitis Symptom and Problem Index; VAS, visual analogue scale.

## Competing interests

The authors declare that they have no competing interests.

## Authors' contributions

TT conceived the study and participated in analyses of the data. All authors assisted in the interpretation of data and approved the final version of the manuscript.

## Pre-publication history

The pre-publication history for this paper can be accessed here:

http://www.biomedcentral.com/1471-2490/11/11/prepub
